# Acidosis and in-hospital mortality in patients with pulmonary hypertension: a retrospective cohort study based on the MIMIC-IV database

**DOI:** 10.3389/fmed.2025.1654432

**Published:** 2025-09-05

**Authors:** Yuheng Ye, Di Yin, Yi Wang, Jiancheng Lin, Jiayan Sun, Xiaowan Wang, Qiang Guo

**Affiliations:** Department of Critical Care Medicine, The Fourth Affiliated Hospital of Soochow University, Suzhou, China

**Keywords:** pulmonary hypertension, acidosis, mortality, association analysis, risk stratification analysis

## Abstract

**Background:**

Pulmonary hypertension (PH) is a life-threatening disease. However, acidosis could be used to predict the prognosis of critically ill patients. Consequently, this study was to identify the link between acidosis and in-hospital death of PH patients based on the Medical Information Mart for Intensive Care-IV (MIMIC-IV) database.

**Methods:**

Eligible subjects from the MIMIC-IV database were selected for this analysis (2008–2019), after which differences in variables between the survival statuses of PH patients were evaluated. Subsequently, employing three weighted multiple logistic regression models to investigate the link between acidosis and PH. Further, risk stratification analysis were applied to explore the relationships between acidosis as well as other covariates and PH.

**Results:**

Total 2,530 PH patients (247 dead and 2,283 live or 157 acidosis and 2,373 non-acidosis) were included in the analysis. Next, the result indicated highly significant differences between the dead and live groups in factors such as acidosis and sepsis (*p* < 0.0001). It also showed highly significant differences between the acidosis and non-acidosis groups in factors such as creatinine and sepsis (*p* < 0.0001). Subsequently, a consistent significant association was found between acidosis and PH, there into, Model 1 displayed an odds ratio (OR) of 5.53 (95% confidence interval (CI): 3.83–7.92, *p* = 2.71 × 10^−20^), Model 2 showed an OR of 5.56 (95% CI: 3.83–8.00, *p* = 6.33 × 10^−20^), Model 3 reported an OR of 2.19 (95% CI: 1.36–3.51, *p* = 1 × 10^−3^), indicating that the impact of acidosis on PH was not significantly affected by other covariates. Notably, risk stratification further revealed acidosis as a risk factor for PH was stable across populations (OR > 1, *p* < 0.05).

**Conclusion:**

This study identified acidosis was a risk factor for PH, highlighting the importance of monitoring in PH patients at risk for acidosis.

## 1 Introduction

Pulmonary hypertension (PH) is a complex clinical condition primarily characterized by an abnormal increase in pulmonary artery pressure (1). Pulmonary vasculopathy, marked by pathological remodeling and vasoconstriction of the pulmonary arteries and, in certain subtypes of pulmonary hypertension, the veins, leads to worsening shortness of breath, reduced exercise capacity, right ventricular (RV) dysfunction, and ultimately, death (2). Dysregulated cellular metabolism, oxidant stress, hypoxia and metabolic signaling, fibrosis, thrombosis, and extracellular matrix remodeling are all considered to play significant roles in the occurrence and progression of pulmonary hypertension (3). Present treatment approaches of PH mainly aim at the signaling pathways of endothelin, nitric oxide, and prostacyclin, centering at oral administration of endothelin receptor antagonists and phosphodiesterase 5 inhibitors (4). According to data from the National Center for Health Statistics, the 1-year survival rate for treated PH is approximately 80%, while for untreated is 60%, dropping to below 35% after 5 years (5). Meanwhile, recent guidelines from the European Society of Cardiology and the European Respiratory Society indicate that delays in diagnosing and treating PH are frequently observed (6). Therefore, a comprehensive identification of the factors influencing the onset of PH can effectively improve its prognosis and clinical outcomes.

Acidemia is frequently observed in critical care units. A blood pH of less than 7.35 or a hydrogen ion concentration exceeding 45 nmol/L is often regarded as acidosis. However, multiple acid–base disorders can occur simultaneously, which may result in the pH may remain within the normal range (7). Hydrogen ions can directly influence vascular smooth muscle, leading to vasoconstriction (8). Additionally, an increase in hydrogen ion concentration can stimulate the proliferation of smooth muscle cells, exacerbating the remodeling of small arteries (9). A study indicated that a decrease in blood pH is associated with increased pulmonary vascular resistance (PVR) in patients with Type I pulmonary hypertension. However, the research team did not directly establish a direct prognostic relationship between acidosis and patients with Type I pulmonary hypertension (10). Although acidosis may play a significant role in PVR and the development of PH, there are currently few clinical studies that focus on the relationship between acidosis and clinical outcomes in patients with pulmonary hypertension (11). Therefore, focusing on exploring the relationship between acidosis and clinical outcomes in PH patients will help clarify the correlation between acidosis and PH, as well as further investigate the specific mechanisms by which acidosis impacts these clinical outcomes, providing new perspectives for treatment approaches to improve the prognosis of PH patients.

MIMIC-IV is an open-source database that offers a comprehensive collection of anonymized clinical data related to ICU patient care, including an enhanced and updated array of clinical variables, encompassing demographic information, detailed physiological data, and therapeutic interventions (12). Huang et al. (13) found a negative correlation between the advanced lung cancer index and all-cause mortality in patients with acute ischemic stroke using the MIMIC-IV database. By utilizing the this database, researchers have demonstrated that a rapid decline in platelet counts serves as a critical prognostic factor in septic patients in the intensive care unit (14). The database provides valuable insights into patient care and their responses to treatments, presenting exciting opportunities for research on various diseases related to critical care.

This study utilized clinical data of PH patients sourced from the MIMIC-IV database and conducted baseline statistical analyses to explore the differences in these variables based on the survival status of different PH patients. To further investigate whether acidosis is a risk factor for patients with PH, we performed association analysis and risk stratification analysis for evaluation. Through this study, we aim to offer scientific evidence and new perspectives for clinical practice in the management of PH.

## 2 Materials and methods

### 2.1 Source of data

In this retrospective study, data on pulmonary hypertension (PH) from the MIMIC-IV,^1^ were used. MIMIC-IV is an open-access database that consolidates de-identified clinical data from ICU patients admitted to Beth Israel Deaconess Medical Center (BIDMC, https://eye.hms.harvard.edu/bidmc) from 2008 to 2019. It should be noted that the BIDMC Institutional Review Board approved the waiver of informed consent and approved the sharing of research resources. Data can be accessed online at: https://physionet.org/content/mimiciv/1.0/. Permission to access and use the MIMIC-IV database for this study was granted by both the Massachusetts Institute of Technology and the Institutional Review Board at Beth Israel Deaconess Medical Center (BIDMC, Boston, MA, United States).

For this study, medical records from the years 2008 to 2019 were utilized to collect data. The inclusion criteria were as follows: (1) we enrolled PH patients based on the International Classification of Diseases (ICD)-9 code 4160, 4168 and ICD-10 code I270, I272, I2720-I2724, I2729; (2) adults ≥ 18 years of age with complete medical records; (3) length of stay in the ICU ≥ 24 h; (4) only first admission data from patients with multiple ICU admissions were included. Next, participants lacking data on covariates should be excluded, thus in this study, indicators with more than 20% missing values were deleted. Average values or median were used to replace missing values when percentage missing was less than 10%. The remaining data (10–20% missing) were obtained by multiple interpolation method. Ultimately, 2,530 PH patients were included, the detailed inclusion and exclusion process was shown in the Table 1.

**Table 1:** Inclusion and exclusion process of PH patients.

Variables	Excluding condition	Number of persons included
Pulmonary hypertension (PH)	Reject and Exclude Missing Values	7,621
Admission to ICU	Reject and Exclude Missing Values	5,145
Admission to ICU ≥ 1 d	Reject and Exclude Missing Values	4,274
Gender	Reject and Exclude Missing Values	4,274
Age ≥ 18	Reject and Exclude Missing Values	4,274
Height, weight	Reject and Exclude Missing Values	2,534
White blood cells (WBCs)	Percentage missing = 0.0019731and Replace missing values with average values	2,534
Hemoglobin	Percentage missing = 0.0015785 and Replace missing values with average values	2,534
Platelets	Percentage missing = 0.0015785 and Replace missing values with average values	2,534
Creatinine	Percentage missing = 0.0015785 and Replace missing values with average values	2,534
blood urea nitrogen (BUN)	Percentage missing = 0.0015785 and Replace missing values with average values	2,534
International normalized ratio (INR)	Percentage missing = 0.0485398 and Replace missing values with average values	2,534
Prothrombin time (PT)	Percentage missing = 0.0485398 and Replace missing values with average values	2,534
Partial thromboplastin time (PTT)	Percentage missing = 0.0513022 and Replace missing values with average values	2,534
Bicarbonate	Percentage missing = 0.0019731 and Replace missing values with average values	2,534
Chloride	Percentage missing = 0.0023677 and Replace missing values with average values	2,534
Calcium	Percentage missing = 0.1535122 and Replace missing values with average values	2,534
Potassium	Percentage missing = 0.0035516 and Replace missing values with average values	2,534
Sodium	Percentage missing = 0.0027624 and Replace missing values with average values	2,534
Heart rate	Percentage missing = 0.0011838 and Replace missing values with average values	2,534
Mean arterial blood pressure(MBP)	Percentage missing = 0.0011838 and Replace missing values with average values	2,534
Respiratory rate (RR)	Percentage missing = 0.0011838 and Replace missing values with average values	2,534
Glucose	No missing data	2,534
Systolic blood pressure(SBP)	Percentage missing = 0.0011838 and Replace missing values with average values	2,534
Partial pressure of carbon dioxide (PCO2)	Percentage missing = 0.1937648 and Multiple interpolation method to obtain data to replace missing values	2,534
Urineoutput	Percentage missing = 0.0264404 and Replace missing values with average values	2,534
Glasgow coma scale(GCS) score	Reject and Exclude Missing Values	2,531
Sequential organ failure assessment (SOFA) score	No missing data	2,531
SOFA-cardiovascular score	No missing data	2,531
SOFA-central nervous system (CNS) score	No missing data	2,531
Logistic organ dysfunction gystem (LODS) score	No missing data	2,531
LODS-neurologic score	No missing data	2,531
LODS-cardiovascular score	No missing data	2,531
LODS-pulmonary score	No missing data	2,531
Mechvent	No missing data	2,531
Acidosis	Reject and Exclude Missing Values	2,530

Indicators with more than 20% missing values were deleted. Indicators between 10 and 20% missing were obtained by multiple interpolation method. Average values or median were used to replace missing values when percentage missing was less than 10%.

### 2.2 Definition variables

The outcome of this study was in-hospital death of PH patients, and acidosis was chosen as exposure factor. Exposure (acidosis) was defined as the lowest arterial blood pH < 7.35 measured within the first 24 h after ICU admission. We selected the first 24-h window to capture the earliest and most severe acid–base derangement, which is less likely to be confounded by subsequent therapeutic interventions and aligns with prior critical care prognostic studies. Furthermore, to explore whether the exposure factor and the outcome were influenced by confounders, the baseline characteristics of the subjects were thus extracted as covariates, including (1) baseline information: gender, age, height, weight, body mass index (BMI); (2) laboratory examination result: mean arterial blood pressure (MBP), white blood cells (WBCs), creatinine, blood urea nitrogen (BUN), hemoglobin, platelets, heart rate, prothrombin time (PT), chloride, sodium, international normalized ratio (INR), calcium, potassium, partial thromboplastin time (PTT), systolic blood pressure (SBP), glucose, respiratory rate (RR), urineoutput, partial pressure of carbon dioxide (PCO2), bicarbonate; (3) system scores: sequential organ failure assessment (SOFA) score, sofa-cardiovascular score, sofa-central nervous system (CNS) score, logistic organ dysfunction gystem (LODS) score, LODS-neurologic score, LODS-cardiovascular score, LODS-pulmonary, scoreglasgow coma scale (GCS) score. (4) clinical intervention: mechvent; (5) comorbidities: diabetes, chronic obstructive pulmonary disease, sepsis, heart failure, pneumonia, urinary tract infection. The variables were classified as either categorical or continuous. All continuous variables were statistically described using medians (upper and lower quartiles). A thorough overview of all covariates and their respective categorization was presented (Supplementary Table 1).

### 2.3 Baseline characteristics

Based on survival status, PH patients were categorized into dead and live groups to investigate potential differences in variables across the two groups. Additionally, to explore the link between different baseline characteristics and acidosis, PH patients were categorized into acidosis (pH < 7.35) and non-acidosis (pH > 7.35) groups based on acidosis exposure.

### 2.4 Association analysis between the acidosis and outcome events

To further analyze the relationship between acidosis and survival in patients with PH, based on included variables, utilizing “survey” R package (v 4.4.2) (15), three weighted multiple logistic regression models were created to compute adjusted OR along with 95% CI. Among the three models, Model 1 was unadjusted which included only PH and acidosis. Model 2 adjusted Model 1 by including additional covariates such as age, gender, height, weight and BMI. Model 3 was further adjusted to include all variables.

### 2.5 Subgroup risk stratification analysis

To validate the stability of the link between acidosis and the risk of PH across diverse populations, subgroup risk stratification analysis was performed for categorical variables (including age, gender, mechvent, diabetes, chronic obstructive pulmonary disease, sepsis, heart failure, pneumonia, and urinary tract infection; grouping details are shown in Table 2) using Model 2. The same analysis was repeated based on Model 3. A forest plot was created using the “forestplot” R package (v 3.1.3) for visual summary of findings (16).

**Table 2:** Grouping variables of subgroup risk stratification analysis.

Variables	Subgroups	
Age	≤ 65 years	> 65 years
Gender	Female	Male
Diabetes	No	Yes
Chronic obstructive pulmonary disease	No	Yes
Sepsis	No	Yes
Heart failure	No	Yes
Pneumonia	No	Yes
Urinary tract infection	No	Yes
Mechvent	No	Yes

These categorical variables of weighted multiple logistic regression models were used in analysis based on Model 2 and Model 3.

### 2.6 Statistical analysis

All statistical analyses were conducted using R software (v 4.2.2). The “Tidyverse” R package (v 2.0.0) was used for data processing (17). For comparing variables across groups (dead vs. live; acidosis vs. non-acidosis), the univariate. Table function in the “Publish” R package (v 2023.1.17, https://cran.r-project.org/web/packages/Publish/index.html) was used for statistical summaries. The Kruskal-Wallis test was applied to compare distributions of non-normally distributed continuous variables, and the weighted Chi-square test was used to evaluate differences in categorical variables (expressed as percentages). Weighted multiple logistic regression models were used for subgroup analysis in Model 2 and Model 3. Continuous variables with a normal distribution were presented as mean ± standard deviation; non-normally distributed continuous variables were reported as median with interquartile range. Categorical variables were summarized as frequencies and percentages. All tests were two-tailed, with statistical significance set at *p* < 0.05.

## 3 Results

### 3.1 Exploration of the differences in variables between different groups

Following the exclusion criteria, 2,530 participants were ultimately incorporated into this study. The baseline characteristics revealed highly significant difference in acidosis between the dead and live groups (*p* < 0.0001). During the hospitalization period, there were 247 deaths, with an overall mortality rate of 9.8% (247/2530). In the acidosis group (157 cases), the in-hospital mortality rate was 33.12% (52/157); in the non-acidosis group (2,373 cases), the in-hospital mortality rate was 8.22% (195/2373) (Table 3). In addition, creatinine, BUN, INR, PT, bicarbonate, chloride, calcium, MBP, RR, glucose, SBP, urineoutput, SOFA score, SOFA-cardiovascular score, LODS score, LODS-cardiovascular score, LODS-pulmonary score and sepsis were also highly significantly different (*p* < 0.0001) between two groups (Table 3). Furthermore, in both the acidosis (157 PH patients) and non-acidosis groups (2,373 PH patients), the baseline characteristics also revealed notable disparities in creatinine, BUN, INR, bicarbonate, chloride, SOFA score, SOFA-cardiovascular score, LODS score, LODS-pulmonary score, and sepsis (p < 0.0001) (Table 4). All these suggested that covariates such as creatinine, BUN, INR, bicarbonate, chloride, SOFA score, SOFA-cardiovascular score, LODS score, LODS-pulmonary score, and sepsis had highly significant effects on both mortality in PH patients and the prevalence of acidosis in patients with PH.

**Table 3:** Baseline characteristics of all patients between dead and alive groups.

Variables	Level	Dead (*n* = 247)	Alive (*n* = 2,283)	Total (*n* = 2,530)	*p* value
Acidosis	Yes	21.1 (*n* = 52)	4.6 (*n* = 105)	6.2 (*n* = 157)	< 0.0001
	No	78.9 (*n* = 195)	95.4 (*n* = 2,178)	93.8 (*n* = 2,373)	
Gender	F	47.8 (*n* = 118)	47.7 (*n* = 1,090)	47.7 (*n* = 1,208)	1.0000
	M	52.2 (*n* = 129)	52.3 (*n* = 1,193)	52.3 (*n* = 1,322)	
Age		74.9 (63.6, 83.8)	71.9 (61.4, 80.8)	72.2 (61.7, 81.1)	0.0007
Weight		73.5 (63.0, 93.2)	80.9 (67.4, 97.0)	80.2 (67.0, 96.7)	0.0004
Height		165 (157, 175)	168 (160, 175)	168 (160, 175)	0.0854
BMI		26.6 (22.9, 32.1)	28.5 (24.5, 33.6)	28.3 (24.3, 33.5)	0.0009
WBCs		10 (6.8, 13.8)	9.2 (6.7, 12.2)	9.2 (6.7, 12.4)	0.0131
Hemoglobin		9.2 (7.9, 10.9)	9.3 (8.1, 11.0)	9.3 (8.0, 10.9)	0.2473
Platelets		156 (95.0, 212.5)	153 (109, 214)	154 (108, 214)	0.2869
Creatinine		1.3 (0.9, 2.2)	1 (0.7, 1.5)	1 (0.8, 1.5)	< 0.0001
BUN		31 (19.5, 47.5)	20 (14, 34)	21 (15, 36)	< 0.0001
INR		1.4 (1.2, 1.7)	1.2 (1.1, 1.4)	1.2 (1.1, 1.4)	< 0.0001
PT		14.9 (13.3, 18.7)	13.7 (12.5, 15.6)	13.9 (12.6, 15.6)	< 0.0001
PTT		31.9 (27.2, 38.0)	30 (26.9, 33.9)	30.1 (26.9, 34.2)	0.0041
Bicarbonate		20 (17, 25)	22 (20, 25)	22 (20, 25)	< 0.0001
Chloride		99 (95, 103)	102 (98, 106)	102 (98, 106)	< 0.0001
Calcium		8.1 (7.5, 8.6)	8.2 (7.9, 8.7)	8.2 (7.9, 8.6)	< 0.0001
Potassium		4 (3.6, 4.5)	4 (3.6, 4.3)	4 (3.6, 4.4)	0.9265
Sodium		137 (133, 140)	137 (135, 139)	137 (134, 139)	0.1044
Heart rate		70 (60, 80)	68 (60, 77)	68 (60, 77)	0.0345
MBP		53 (46, 60)	57 (51, 63)	56.5 (50, 62)	< 0.0001
RR		13 (10, 16)	12 (10, 14)	12 (10, 14)	< 0.0001
glucose		112 (89, 139)	98 (84, 118)	99 (84, 119)	< 0.0001
SBP		82 (74.0, 92.5)	87 (79, 96)	87 (79.0, 95.4)	< 0.0001
PCO2		36 (31.0, 40.5)	36.6 (32, 38)	36.6 (32, 38)	0.5931
Urineoutput		1,040 (410, 1,925)	1,660 (1,050, 2,435)	1,628.5 (995, 2,405)	< 0.0001
GCS score		15 (13, 15)	15 (14, 15)	15 (14, 15)	0.1629
SOFA score		8 (5, 11)	5 (3, 8)	5 (3, 8)	< 0.0001
SOFA-cardiovascular score		2 (1, 4)	1 (1, 3)	1 (1, 3)	< 0.0001
SOFA-CNS score		0 (0, 1)	0 (0, 1)	0 (0, 1)	0.2585
LODS score		8 (6, 10)	5 (3, 7)	5 (3, 7)	< 0.0001
LODS-neurologic score		0 (0, 1)	0 (0, 0)	0 (0, 0)	0.0051
LODS-cardiovascular score		1 (0, 1)	1 (0, 1)	1 (0, 1)	< 0.0001
LODS-pulmonary score		3 (0, 3)	1 (0, 3)	1 (0, 3)	< 0.0001
Mechvent	Yes	56.3 (*n* = 139)	54.6 (*n* = 1,246)	54.7 (*n* = 1,385)	0.6585
	No	43.7 (*n* = 108)	45.4 (*n* = 1,037)	45.3 (*n* = 1,145)	
Diabetes	Yes	35.6 (*n* = 88)	41.9 (*n* = 956)	41.3 (*n* = 1,044)	0.0678
	No	64.4 (*n* = 159)	58.1 (*n* = 1,327)	58.7 (*n* = 1,486)	
Chronic obstructive pulmonary disease	Yes	25.5 (*n* = 63)	26.5 (*n* = 605)	26.4 (*n* = 668)	0.7943
	No	74.5 (*n* = 184)	73.5 (*n* = 1,678)	73.6 (*n* = 1,862)	
Sepsis	Yes	44.9 (*n* = 111)	24.5 (*n* = 560)	26.5 (*n* = 671)	< 0.0001
	No	55.1 (*n* = 136)	75.5 (*n* = 1,723)	73.5 (*n* = 1,859)	
Heart failure	Yes	71.3 (*n* = 176)	74.9 (*n* = 1,710)	74.5 (*n* = 1,886)	0.2409
	No	28.7 (*n* = 71)	25.1 (*n* = 573)	25.5 (*n* = 644)	
Pneumonia	Yes	42.5 (*n* = 105)	35.1 (*n* = 801)	35.8 (*n* = 906)	0.0250
	No	57.5 (*n* = 142)	64.9 (*n* = 1,482)	64.2 (*n* = 1,624)	
Urinary tract infection	Yes	23.5 (*n* = 58)	32.2 (*n* = 734)	31.3 (*n* = 792)	0.0066
	No	76.5 (*n* = 189)	67.8 (*n* = 1,549)	68.7 (*n* = 1,738)	

Values are described by number (%) or median (interquartile range).

**Table 4:** Baseline information of all patients between acidosis and non-acidosis groups.

Variables	Level	Acidosis (*n* = 157)	Non-Acidosis (*n* = 2,373)	Total (*n* = 2,530)	*p* value
Gender	F	43.3 (*n* = 68)	48.0 (*n* = 1,140)	47.7 (*n* = 1,208)	0.2863
	M	56.7 (*n* = 89)	52.0 (*n* = 1,233)	52.3 (*n* = 1,322)	
Age		71.6 (61.8, 80.4)	72.3 (61.7, 81.1)	72.2 (61.7, 81.1)	0.7236
Weight		80.5 (66.4, 92.5)	80.1 (67.0, 96.9)	80.2 (67.0, 96.7)	0.3257
Height		168 (160, 173)	168 (160, 175)	168 (160, 175)	0.1942
BMI		28.1 (23.7, 32.6)	28.4 (24.3, 33.5)	28.3 (24.3, 33.5)	0.5490
WBCs		10.4 (7.0, 14.5)	9.2 (6.7, 12.3)	9.2 (6.7, 12.4)	0.0177
Hemoglobin		9.3 (7.7, 10.9)	9.3 (8.1, 10.9)	9.3 (8.0, 10.9)	0.1125
Platelets		154 (107, 195)	154 (108, 215)	154 (108, 214)	0.3495
Creatinine		1.5 (0.9, 2.6)	1 (0.7, 1.5)	1 (0.8, 1.5)	< 0.0001
BUN		29 (18, 51)	21 (15, 35)	21 (15, 36)	< 0.0001
INR		1.4 (1.2, 1.6)	1.2 (1.1, 1.4)	1.2 (1.1, 1.4)	< 0.0001
PT		14.8 (12.9, 17.2)	13.8 (12.5, 15.6)	13.9 (12.6, 15.6)	0.0010
PTT		30.5 (26.3, 34.9)	30.1 (27.0, 34.2)	30.1 (26.9, 34.2)	0.9484
Bicarbonate		18 (15, 21)	22 (20, 25)	22 (20, 25)	< 0.0001
Chloride		99 (95, 103)	102 (98, 106)	102 (98, 106)	< 0.0001
Calcium		8.2 (7.8, 8.6)	8.2 (7.9, 8.7)	8.2 (7.9, 8.6)	0.0142
Potassium		4.1 (3.7, 4.6)	4 (3.6, 4.3)	4 (3.6, 4.4)	0.0086
Sodium		136 (133, 139)	137 (134, 139)	137 (134, 139)	0.1005
Heart rate		68 (59, 82)	68 (60, 77)	68 (60, 77)	0.4870
MBP		56 (49, 63)	56.5 (50, 62)	56.5 (50, 62)	0.4345
RR		13 (10, 15)	12 (10, 14)	12 (10, 14)	0.0128
Glucose		112 (84, 138)	98 (84, 118)	99 (84, 119)	0.0048
SBP		84 (75, 91)	87 (79, 96)	87 (79.0, 95.4)	0.0021
PCO2		34 (29, 38)	36.6 (32, 38)	36.6 (32, 38)	0.0017
Urineoutput		1,300 (650, 2,155)	1,650 (1,012, 2,420)	1,628.5 (995, 2,405)	0.0003
GCS score		15 (14, 15)	15 (14, 15)	15 (14, 15)	0.6316
SOFA score		8 (5, 10)	5 (3, 8)	5 (3, 8)	< 0.0001
SOFA-cardiovascular score		3 (1, 4)	1 (1, 3)	1 (1, 3)	< 0.0001
SOFA-CNS score		0 (0, 1)	0 (0, 1)	0 (0, 1)	0.4532
LODS score		7 (5, 10)	5 (3, 7)	5 (3, 7)	< 0.0001
LODS-neurologic score		0 (0, 0)	0 (0, 0)	0 (0, 0)	0.4783
LODS-cardiovascular score		1 (0, 1)	1 (0, 1)	1 (0, 1)	0.0071
LODS-pulmonary score		3 (0, 3)	1 (0, 3)	1 (0, 3)	< 0.0001
Mechvent	Yes	56.7 (*n* = 89)	54.6 (*n* = 1,296)	54.7 (*n* = 1,385)	0.6725
	No	43.3 (*n* = 68)	45.4 (*n* = 1,077)	45.3 (*n* = 1,145)	
Diabetes	Yes	46.5 (*n* = 73)	40.9 (*n* = 971)	41.3 (*n* = 1,044)	0.1966
	No	53.5 (*n* = 84)	59.1 (*n* = 1,402)	58.7 (*n* = 1,486)	
Chronic obstructive pulmonary disease	Yes	31.8 (*n* = 50)	26.0 (*n* = 618)	26.4 (*n* = 668)	0.1325
	No	68.2 (*n* = 107)	74.0 (*n* = 1,755)	73.6 (*n* = 1,862)	
Sepsis	Yes	52.2 (*n* = 82)	24.8 (*n* = 589)	26.5 (*n* = 671)	< 0.0001
	No	47.8 (*n* = 75)	75.2 (*n* = 1,784)	73.5 (*n* = 1,859)	
Heart failure	Yes	77.7 (*n* = 122)	74.3 (*n* = 1,764)	74.5 (*n* = 1,886)	0.3984
	No	22.3 (*n* = 35)	25.7 (*n* = 609)	25.5 (*n* = 644)	
Pneumonia	Yes	40.8 (*n* = 64)	35.5 (*n* = 842)	35.8 (*n* = 906)	0.2110
	No	59.2 (*n* = 93)	64.5 (*n* = 1,531)	64.2 (*n* = 1,624)	
Urinary tract infection	Yes	26.1 (*n* = 41)	31.6 (*n* = 751)	31.3 (*n* = 792)	0.1741
	No	73.9 (*n* = 116)	68.4 (*n* = 1,622)	68.7 (*n* = 1,738)	

Values are described by number (%) or median (interquartile range).

### 3.2 Significant correlation between acidosis and PH

Subsequently, the results of association analysis indicated a consistent and significant correlation between acidosis with PH regardless of the adjustments made to the covariates (*p* < 0.05), among them, Model 1 displayed an OR = 5.53 (95% CI, 3.83–7.92), *p* = 2.71 × 10^−20^, Model 2 showed an OR of 5.56 (95% CI = 3.83–8.00, *p* = 6.33 × 10^−20^), and Model 3 reported an OR of 2.19 (95% CI = 1.36–3.51, *p* = 1.16 × 10^−3^) (Table 5). All these suggested that the impact of acidosis on PH was not significantly influenced by other covariates, highlighting the potential importance of monitoring and managing PH in patients with or at risk for acidosis.

**Table 5:** Association between exposure to acidosis and in-hospital mortality in patients with PH.

Mode	Odds ratio (OR)	95% confidence interval (CI)		*p* value
Model 1	5.53	3.83	7.92	2.71 × 10^−20^
Model 2	5.56	3.83	8.00	6.33 × 10^−20^
Model 3	2.19	1.36	3.51	1.16 × 10^−3^

Model 1, 2 and 3 are three weighted multiple logistic regression models created to compute adjusted OR along with 95% CI. Model 1 included merely PH and acidosis. Model 2 was adjusted based on model 1 by including additional covariates. Model 3 was further adjusted to include all variables.

### 3.3 Acidosis as potential risk factors in different subgroups of PH patients

**Figure 1:**
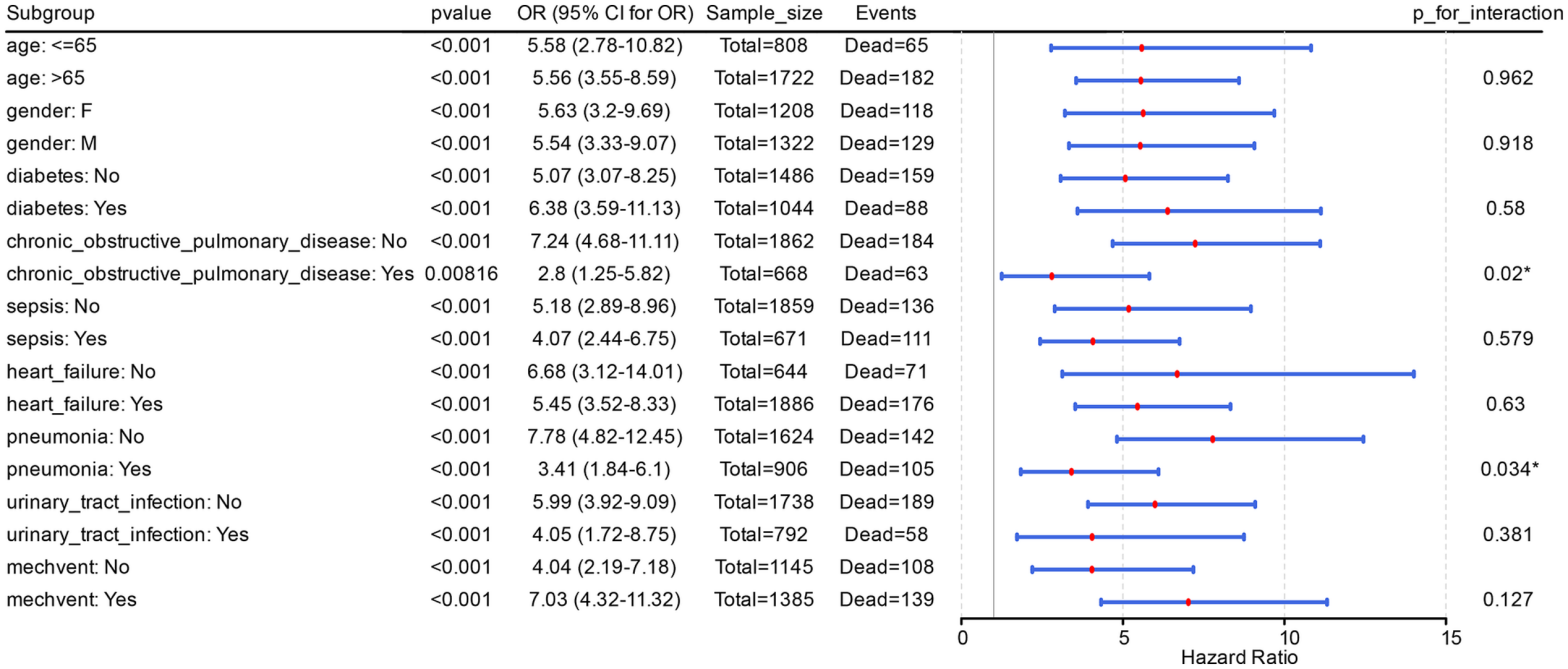
Acidosis is a potential risk factor in different subgroups of PH patients. The figure displayed the odds ratios (ORs) and their corresponding 95% confidence intervals (CIs) for each subgroup, while also annotating the sample size (Sample_size, representing the total number of PH patients in the subgroup) and the number of events (Events, denoting the count of in-hospital deaths among PH patients within the subgroup).

The results of the risk-stratification analysis based on Model 2 showed that, in the vast majority of subgroups, acidosis was a stable predictor of the risk of in-hospital death in all PH patients (*p* < 0.001). Meanwhile, acidosis was a risk factor in different subgroups of PH patients (OR > 1). It was noted that there were significant (P for interaction < 0.05) interactions of acidosis with pneumonia and chronic obstructive pulmonary disease in Model 2 (Figure 1).

**Figure 2:**
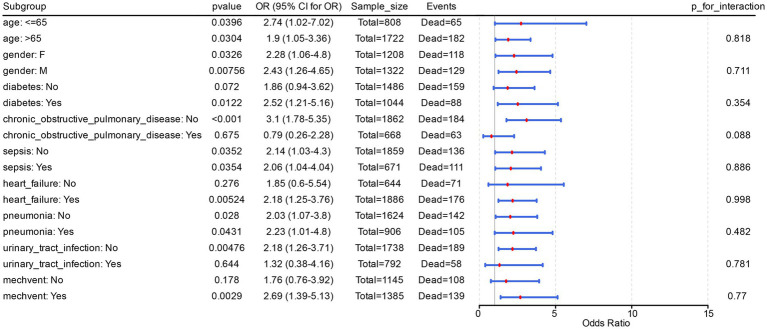
Interaction between acidosis and the other covariant. This figure showed the odds ratio (OR) and 95% confidence interval (CI) for each subgroup, and labeled the sample size (Sample_size, total number of PH patients in the subgroup) and number of events (Events, number of in-hospital deaths among PH patients in the subgroup).

Further, risk stratification analysis in Model 3 clearly demonstrated that acidosis was a risk factor for PH patients with diabetes (*p* = 0.012, OR = 2.52, 95% CI = 1.21–5.16). The same occurred in PH patients with heart failure complications (*p* = 0.005, OR = 2.18, 95% CI = 1.25–3.76), and mechvent (*p* = 0.003, OR = 2.69, 95% CI = 1.39–5.13). Additionally, acidosis was significantly recognised as a risk factor in PH patients of different ages and sexes, with or without complications of sepsis, and with or without complications of pneumonia (OR > 1, *p* < 0.05). Finally, in Model 3, there was no significant (*p* > 0.05) interaction between acidosis and the other covariant (Figure 2). All these suggested that acidosis as a risk factor is stable in different subgroups of PH patients.

## 4 Discussion

PH is indeed a serious and life-threatening condition characterized by abnormally elevated pressure in the pulmonary arteries, which can lead to right heart failure and increased mortality (18). Five primary categories of pulmonary hypertension are identified, and in all its variations, it is linked to detrimental vascular remodeling, characterized by obstruction, rigidity, and vasoconstriction of the pulmonary blood vessels (19). An increase in hydrogen ion concentration may increase cellular calcium exchange and mediate calcium release,leading to smooth muscle contraction and an increased PVR (20). Meanwhile, the change in hydrogen ion concentration may effect on vascular proliferation (21). Our study found that acidemia is a risk factor for in-hospital mortality in patients with PH according to the recent 2022 ESC/ERS guidelines (6).

Sepsis, left heart disease, and pulmonary diseases are common causes of death in PH patients (22). In our study, baseline characteristics showed significant differences in sepsis and SOFA cardiovascular scores between the death and the survival groups. He et al. (23) found that cardiac injury index in sepsis patients with PH was higher than in those without PH, based on the MIMIC-III database. Price et al. (24) found that severe PH patients experience a significant decrease in cardiac output due to right heart failure. This phenomenon can be explained by the unique pathophysiological mechanism of right heart failure. The body compensates or only requires low-dose vasoactive drugs to maintain baseline blood pressure, resulting in a decrease in SOFA cardiovascular score (dependent on vasoactive drug dosage and blood pressure values) (25). Our research found a lower SOFA cardiovascular score in more severely affected PH patients, which, to some extent, is consistent with their findings. This may suggest that the SOFA cardiovascular score may not fully reflect the severity of the condition in critical patients with PH, and should be comprehensively evaluated in conjunction with indicators such as BNP and echocardiography. Additionally, our analysis results showed a significant baseline difference in sepsis between PH patients and controls. This may be due to the fact that PH can impair cardiac function in patients, affecting the prognosis of sepsis (26). At the same time, sepsis can exacerbate pulmonary hypertension by promoting vascular remodeling through inflammatory responses and immune cell infiltration (27). We found that ICU patients with acidemia had higher blood urea nitrogen (BUN) levels. Existing reports have indicated a close association between BUN and the prognosis of PH (18, 28). Higher PVR increases the clinical risk of death and heart failure in patients with elevated pulmonary artery pressure (29). This also helps to explain our findings on the relationship between acidosis and PH to some extent. Our study indicated that in PH patients who have complications from diabetes and heart failure, acidemia is a risk factor for in-hospital mortality. This may be because diabetes often leads to cardiac dysfunction, which can increase the burden on the right heart and subsequently lead to or worsen PH (30). Cardiac dysfunction can lead to inadequate tissue perfusion, resulting in lactic acid accumulation and metabolic acidosis. It may also increase the workload on the right heart, further exacerbating the development of PH (8). Additionally, insulin resistance in diabetic patients may negatively impact pulmonary vascular function (31). The hyperglycemic state associated with diabetes can lead to microvascular damage, which affects the function of pulmonary microvessels. This damage may result in vascular remodeling and increased pressure within the pulmonary circulation (32). Patients with diabetes, especially those experiencing diabetic ketoacidosis or hyperglycemic hyperosmolar state, may develop metabolic acidosis. This condition may lead to a decrease in blood pH, which can affect the function of vascular smooth muscle, potentially causing pulmonary vasoconstriction and increasing pulmonary artery pressure (33). Chronic inflammation is common in patients with diabetic and heart failure (34). Inflammatory mediators can promote pulmonary vascular remodeling, leading to dysfunction and causing vasoconstriction (35). This, in turn, exacerbates pulmonary hypertension (PH) and contributes to its further deterioration (36). This may also explain the differences in leukocyte test results in pulmonary hypertension patient groups with varying clinical outcomes.

Furthermore, this study found that obesity, age, and INR may be risk factors influencing clinical outcomes in patients with pulmonary hypertension, which have been reported in the literature (37–39). However, due to individual differences, evaluating the prognosis of HP patients based on these characteristics is difficult and inaccurate. Therefore, this study explored the correlation between acidosis and PH from the perspective of risk association, which has more reference significance for effectively evaluating the prognosis of PH patients.

Moreover, we found that elevated serum calcium levels may be a risk factor influencing mortality in patients with pulmonary hypertension. This could be due to excessively high levels of calcium affecting the contraction of small to medium-sized arterial smooth muscle, thereby exacerbating the progression of pulmonary hypertension (40). By improving our understanding of how these risk factors act individually and collectively in PH development, we can more effectively identify and manage high-risk populations, thereby improving the clinical outcomes of PH. Our study constructed a baseline statistical table to explore factors with significant differences between the deceased and surviving groups, as well as between the acidemia and non-acidemia groups. Through correlation and risk stratification analyses, we found that the impact of acidemia on in-hospital mortality in PH patients was not confounded by other covariates. Acidemia demonstrated stable predictive value for in-hospital mortality risk across different subpopulations of PH patients.

However, this study also has its limitations. This study is based on the MIMIC-IV database and only included pulmonary arterial hypertension (PH) patients admitted to the ICU. The data was only sourced from a single center at Beth Israel Deaconess Medical Center (BIDMC) in the United States, which may have selection bias due to regional differences in medical practice and patient population characteristics. In addition, the diagnostic code (ICD-9/10) for PH in the MIMIC-IV database did not clearly distinguish subtypes, resulting in the study’s conclusions not reflecting subtype specificity. Therefore, future research needs to conduct multicenter, prospective cohort studies, combined with detailed classification of PH subtypes and more comprehensive clinical data, to validate the conclusions of this study and explore its potential mechanisms. Due to the dataset being sourced from a publicly available database, there may be a certain degree of heterogeneity that could affect the interpretation of the results. This heterogeneity may arise from variations in clinical practices across different sections, the diversity of patient backgrounds, and differences in data collection methods. Therefore, there may be potential biases and limitations that require careful consideration to ensure the reliability and applicability of the conclusions. Despite the fact that there is evidence indicating that an elevation in proton concentration could increase PVR, few studies have been conducted to elucidate the relationship between acidemia and pulmonary hypertension (41). Further studies are required to help clarify the underlying mechanisms linking acidemia to the pathophysiology of PH, as well as assess the potential impact of acid–base balance on PH patient outcomes.

Our findings identified that the presence of acidemia consistently correlates with an increased risk of mortality during hospitalization in PH patients. However, establishing a causal relationship requires further investigation.

## Data Availability

The raw data supporting the conclusions of this article will be made available by the authors, without undue reservation.

## Footnotes

^1^ https://mimic.mit.edu/docs/iv/
